# Neurobiological Correlates of Fatherhood During the Postpartum Period: A Scoping Review

**DOI:** 10.3389/fpsyg.2022.745767

**Published:** 2022-02-03

**Authors:** Mónica Sobral, Francisca Pacheco, Beatriz Perry, Joana Antunes, Sara Martins, Raquel Guiomar, Isabel Soares, Adriana Sampaio, Ana Mesquita, Ana Ganho-Ávila

**Affiliations:** ^1^Faculty of Psychology and Educational Sciences, Center for Research in Neuropsychology and Cognitive Behavioral Intervention, University of Coimbra, Coimbra, Portugal; ^2^Psychology Research Centre (CIPsi), School of Psychology, University of Minho, Braga, Portugal

**Keywords:** fatherhood, neuroplasticity, neuroendocrine, molecular mechanisms, neuroimaging correlates, postpartum

## Abstract

During the postpartum period, the paternal brain suffers extensive and complex neurobiological alterations, through the experience of father–infant interactions. Although the impact of such experience in the mother has been increasingly studied over the past years, less is known about the neurobiological correlates of fatherhood—that is, the alterations in the brain and other physiological systems associated with the experience of fatherhood. With the present study, we aimed to perform a scoping review of the available literature on the genetic, neuroendocrine, and brain correlates of fatherhood and identify the main gaps in the current knowledge. PubMed, Scopus, and Web of Science electronic databases were searched for eligible studies on paternal neuroplasticity during the postpartum period, over the past 15 years. Reference lists of relevant key studies and reviews were also hand-searched. The research team independently screened the identified studies based on the established inclusion criteria. Extracted data were analyzed using tables and descriptive synthesis. Among the 29 studies that met our inclusion criteria, the vast majority pertained to neuroendocrine correlates of fatherhood (*n* = 19), followed by brain activity or connectivity (*n* = 7), association studies of candidate genes (*n* = 2), and brain structure correlates (*n* = 1). Collectively, studies published during the past 15 years suggest the existence of significant endocrine (testosterone, oxytocin, prolactin, and cortisol levels) and neurofunctional alterations (changed activity in several brain networks related to empathy and approach motivation, emotional processing and mentalizing, emotion regulation, dorsal attention, and default mode networks) as a result of fatherhood, as well as preliminary evidence of genetic variability accounting for individual differences during the postpartum period in fathers. No studies were so far published evaluating epigenetic mechanisms associated with the paternal brain, something that was also the focus of the current review. We highlight the need for further research that examines neuroplasticity during the experience of fatherhood and that considers both the interplay between hormones and simultaneous assessment of the different biomarkers (e.g., associations between hormones and neural activity); data collection protocols and assessment times should also be refined.

## Introduction

The transition to parenthood represents a transformative period, marked by diverse co-occurring biological, psychological, social, economic, and behavioral changes (e.g., decreasing levels of testosterone, increasing levels of oxytocin, changes in thoughts and behaviors oriented toward the infant; Bakermans-Kranenburg et al., 2019). These changes seem to facilitate experience-dependent acquisitions of parenting skills, aimed at caring for the infant and securing survival (Swain et al., 2014). Consequently, this period may be considered a potential “critical window” for neuroplasticity, alongside infancy and adolescence (Saxbe et al., 2018). Although a substantial amount of evidence about motherhood biomarkers has surfaced, fatherhood has been less researched by comparison. Herein, we aim to conduct a scoping review of the available literature on alterations in several neurobiological correlates underlying the experience of fatherhood.

Neuroplasticity refers to the reorganization of the brain (physiological and anatomic changes) as a result of our interactions with the environment, allowing for the adaptation to new circumstances and demands (Demarin et al., 2014). A reflection of neuroplasticity processes is the thickness or thinning of the gray matter architecture of the brain (refining, severing, or forging of neural connections; Saxbe et al., 2018). During pregnancy and the postpartum period, the parental brain suffers alterations in its structure and function. In fathers, these alterations mostly occur during the postpartum period, through the experience of father–infant interactions, and may correspond to increased neuroplasticity (e.g., Kim et al., 2014). Despite not experiencing in-person the hormonal changes of pregnancy, research has shown that fathers engage similar neural circuitry when processing infant-related stimuli compared to mothers (Swain et al., 2007). Additionally, fathers undergo significant neuroendocrine alterations during the transition to parenthood that have been found to be associated with greater involvement in caregiving (e.g., longitudinal decreases in testosterone; Gettler et al., 2011).

Furthermore, intrinsic individual genetic characteristics, such as subtle changes in the DNA sequence, called single nucleotide polymorphisms (SNPs), might contribute to differences in parental responsiveness. Indeed, studies with mothers show that variability in parenting styles can be moderated by polymorphic variations in target genes associated with affiliative and reward systems. Particularly relevant in this regard is the oxytocinergic pathway, which includes the oxytocin neuropeptide (OT) and the oxytocin receptor (OXTR). Generically, genetic variability in the OXTR gene has been associated with sociability, caregiving, and parenting (Feldman et al., 2016; Feldman, 2017), with particular evidence showing that the GG risk genotype of the rs2254298 SNP is associated with autism and depression (Lerer et al., 2008; Costa et al., 2009) and the TT risk genotype of the rs1042778 SNP correlates with lower empathy and prosocial behavior in healthy adults (Israel et al., 2009; Wu et al., 2012), while rs53576 GG genotype is associated with more sensitive parenting and the G allele of the rs1042778 SNP with positive parenting in mothers (Bakermans-Kranenburg and Van Ijzendoorn, 2008; Michalska et al., 2014). Moreover, in Riem et al. (2011) the authors showed that women carrying the presumably protector genotype (GG) were more reactive to the infants’ cry (measured through heart rate response), although women with depression symptoms with the same genotype did not show this reactivity, highlighting the relevance of addressing parenthood and quality of care within a gene-by-environment framework (GxE). Yet, little is known about these genetic mechanisms concerning fathers. Furthermore, epigenetic mechanisms such as DNA methylation (DNAm) have recently entered the research field of parenting behavior. DNAm occurs at the CpG sites of the gene, where methyl residues (CH3) are added. The addition of these molecules attenuates the binding of transcription elements on the gene, which in turn contribute to the lower expression or “silencing” of the gene activity. Indeed, recent research shows that DNAm of the OT gene changes brain volume in important areas for parenting behavior in mothers (i.e., right inferior temporal gyrus; Hiraoka et al., 2021) and that DNAm profile changes during pregnancy predict postnatal variability in maternal behavior (more specifically, in intrusive behavior; Toepfer et al., 2019).

Although growing neuroimaging, molecular and hormonal-based literature has explored the neurobiological basis of parenthood, the majority of research has been conducted on human mothers during pregnancy or the postpartum period (e.g., Kim et al., 2010), and less is known about the specific correlates of the experience of fatherhood. And whereas some mechanisms appear to be shared between mothers and fathers, important differences might exist, possibly driven by biological (e.g., testosterone levels) and experience-related factors (e.g., being a primary caregiver).

Diverse reviews on fatherhood focus on different correlates and measures, such as neuroendocrine (e.g., Storey et al., 2020) and functional neuroimaging (e.g., Swain et al., 2014). Our review aims to further tap into this knowledge, providing a structured, comprehensive, and descriptive search of the literature on the neurobiological correlates of fatherhood associated with postpartum parental caregiving experiences, while extending to molecular and structural brain correlates and identifying the main gaps in the current knowledge. Thus, the following questions will be addressed:

A. What is known about the molecular mechanisms (genetic and epigenetic) related to fatherhood?
B. What is known about the neuroendocrine correlates of fatherhood?
C. What is known about the neurostructural correlates of fatherhood?
D. What is known about the neurofunctional correlates of fatherhood?

## Materials and Methods

Our review aim was too broad to be addressed by a systematic review; instead, it was thought to be better addressed by looking at the scope and nature of the research pertaining to the neurobiological correlates of fatherhood, while identifying research gaps (see guidance by Munn et al., 2018). Based on these objectives, a scoping review was considered to be the most appropriate review strategy. Scoping reviews follow the same structured process as systematic reviews (Munn et al., 2018), however, a formal assessment of the methodological quality of the included studies is generally not performed (Colquhoun et al., 2014; Peters et al., 2020). The review process followed the Preferred Reporting Items for Systematic reviews and Meta-analyses extension for Scoping Reviews (Tricco et al., 2018) for conducting and reporting the results. The review was pre-registered in Open Science Framework.^1^

### Eligibility Criteria

We included full-text primary research studies published in peer-reviewed journals within the last 15 years, given the technological progress of neuroimaging methods. Studies were eligible if they included data from human heterosexual fathers (age range 18–60) and assessed outcomes from infant birth to 1 year postpartum; assessed neuroendocrine (testosterone, oxytocin, prolactin, cortisol levels and manipulations), brain structural changes (gray matter volume and cortical thickness), brain functional changes (task-based patterns of functional activity and connectivity), and/or molecular mechanisms (genetic and epigenetic) among fathers; and were written in English language. The limited timeframe (0–12 months) was selected based on the focus of the review (neurobiological correlates of fatherhood associated with postpartum experiences).

Psychiatric or neurological disorders (in both father and infant) and premature birth were excluded, as the focus of the review was on normative correlates associated with fatherhood. Older adults were excluded as well (>60 years old), given the existing negative association between age and structural alterations in healthy aging (e.g., Salat et al., 2004). We excluded opinion pieces, editorials, conference abstracts, qualitative studies, and reviews.

### Information Sources and Search Strategy

Searches were performed in PubMed, Scopus, and Web of Science electronic databases. Following the Joanna Briggs Institute methodology (Peters et al., 2020), a three-step strategy was performed. Firstly, a limited search of the PubMed database took place, with the following MeSH terms: “(Father OR fatherhood OR men) AND (Brain OR anatomical OR structural OR functional OR connectivity OR activation OR resting state OR neural OR hormonal OR neuroendocrine OR genetic OR epigenetic) AND (postpartum OR postnatal OR perinatal).” Secondly, keywords included in the title and abstract of retrieved papers and the index terms were analyzed. Afterward, a second search was performed across the included databases with the identified and relevant keywords and index terms. The searches were conducted between November and December 2020. In addition to these databases, we hand-searched reference lists of key studies included in our search and reference lists in key reviews published in the field. The full search strategies for all databases are attached as Supplementary Material.

### Selection of Sources of Evidence

Screening was conducted in the Rayyan QCRI software (Ouzzani et al., 2016), where authors FP and MS removed the duplicates across databases. The same two reviewers independently performed the double-screening of titles and abstracts, as well as the full-text articles. A third reviewer was available to resolve inter-rater disagreements when necessary. Inter-rater agreement on study selection was calculated using Cohen’s kappa coefficient (Landis and Koch, 1977). The inter-rater agreement was *k* = 0.86, indicating almost perfect agreement.

### Data Charting Process and Data Items

The included reports were split across two teams of reviewers responsible for extracting data on different neurobiological correlates. FP and MS extracted structural and functional data, while JA, SM, and BP extracted data concerning neuroendocrine and genetic/epigenetic measures. The team cross-reviewed each other’s extraction. After comparing each reviewer’s charted data, disagreements were resolved through discussion and a third reviewer when needed as well. A data charting form was developed *a priori*, including the following information: author name, year of publication, study design, participant characteristics, methodology, and key findings. Individual data forms were constructed for the different measures available.

### Synthesis of Results

In accordance with scoping review guidelines (Tricco et al., 2018; Peters et al., 2020), an assessment of the methodological quality or risk of bias of the included studies was not performed. The available data were organized in a comprehensive system of the diverse measures available. A narrative and tabular synthesis of the extracted data was performed.

## Results

### Selection of Sources of Evidence

**Figure 1:**
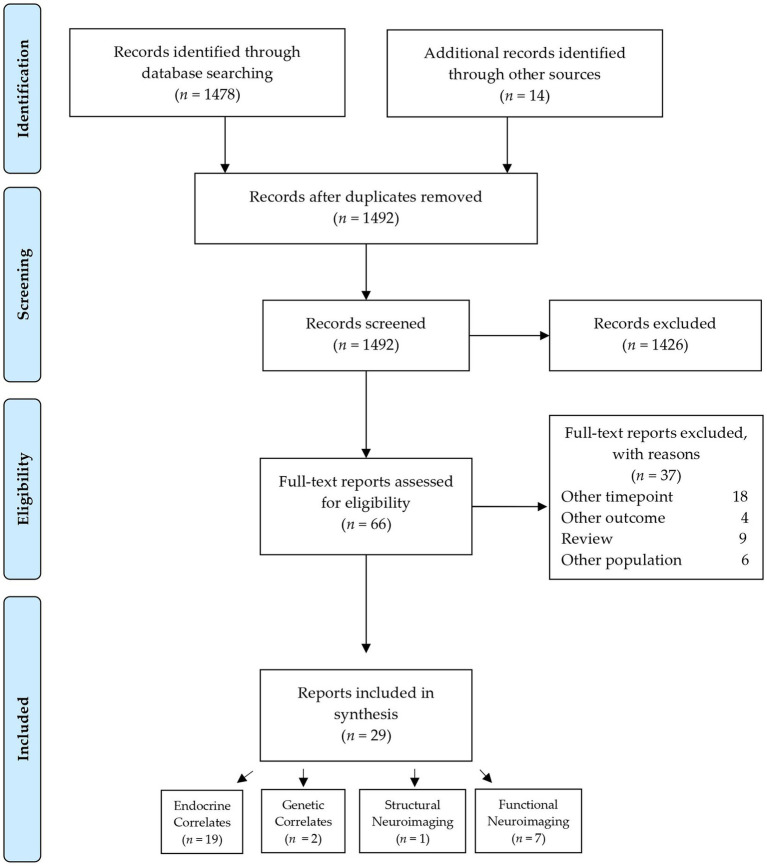
Flow chart of the selection of sources of evidence.

The selection process of sources of evidence was adapted from the Preferred Reporting Items for Systematic Reviews and Meta-Analysis flow diagram. After the removal of duplicates, 1,492 citations were reviewed. Of these, 1,426 were excluded based on the title and abstract. Of the remaining 66 full-text articles assessed for eligibility, 37 were excluded for different reasons, detailed in the Flow Chart in Figure 1. Subsequently, 29 articles were included in this review.

### Characteristics of Sources of Evidence and Synthesis of Results

Details of each study are provided in Table 1. The summary below focuses on key characteristics and findings, organized according to the parameter in question.

**Table 1:** Main characteristics and findings of the studies.

Authors	Father’s sample size	Infant’s age Mean (SD)	Aim	Study design	Assessment of parenting	Analysis	Main findings
**SNPs and hormones**							
Feldman et al. (2012)	*n* = 121	5.78 months (1.13)	To assess whether CD38 and OXTR risk alleles are associated with low plasma OT and less parental touch and gaze synchrony.	Cross-sectional	Coding of Parent–Infant Interaction (parent gaze, child gaze and parent touch)	OT determination: ELISA OXTR and CD38 SNPs determination: SNaPshot Method OXTR rs2254298 (risk allele = G) OXTR rs1042778 (risk allele = T) CD38 SNP rs3796863 (risk genotype = CC)	**SNP rs379686 of the CD38 gene** C allele was associated with lower plasma OT compared with carriers of the A allele. SNP rs2254298 and rs1042778 of the OXTR gene. GG and TT, genotype were associated with lower plasma OT compared with individuals carrying the A allele and the G allele, respectively, Reduced plasma OT and both TT genotype (rs1042778) and C allele (rs379686) are related to less parental touch. The interaction of high plasma OT and A allele of the rs379686 SNP of the CD38 gene predicted longer durations of parent–infant gaze synchrony.
Feldman et al. (2013)	*n* = 160 parents (80 couples) at T1 *n* = 128 parents (62 fathers) at T2	T1: 1 month old T2: 6 months old	To understand if parents’ peripheral OT levels and reciprocal parenting style would be individually stable over time and whether it correlates with more optimal allelic variations on OXTR and CD38 genes; To assess if a gene-by-environment interaction occurred, that is, if more optimal allelic variations on the parents’ CD38 gene interacts with early parenting to shape children’s OT response.	Prospective longitudinal	Coding of Parent–Infant Interaction (Gaze, Affect, Vocalizations, Touch)	OT determination: ELISA OXTR and CD38 SNPs: SNaPshot Method OXTR rs2254298 (risk genotype = GG); rs1042778 (risk genotype = TT) CD38 SNP rs3796863 (risk genotype = CC)	**SNP rs3796863 of the CD38 gene** Fathers carriers of CC genotype showed lower peripheral OT. High OT was associated with early parental care.
**Hormones**							
Delahunty et al. (2007)	*n =* 21 couples	T1: 2–3 weeks old T2: 2 months old	To investigate sex differences in hormonal reactivity and to test whether sex-specific developmental trajectory or differences in recent/lifetime levels of exposure to infants influence these differences.	Longitudinal	Questionnaires (previous time with children; number of older and younger siblings; checklist of emotional responses to infant cries; infant contact before testing) Close contact with infant (30 min)	PRL; Plasma; AutoDELFIATM Prolactin kit	Higher increase in PRL in fathers when holding their second child (vs. first child) Fathers showed a non-significant trend toward greater percentage increases in PRL associated with above average child contact. Fathers who spent less than 3 h at home with their newborn prior to the 2–3 weeks postpartum showed significantly greater increase in PRL after holding their babies during than fathers who were home all day with their infants.
							At 2 months postpartum, fathers who held their babies for less than an hour in the 4 h prior testing showed higher baseline PRL concentrations than fathers who held their babies for more than an hour.
Gordon et al. (2010a)	*n* = 37	T1 = 6.97 weeks (2.35) T2 = 25.49 weeks (4.61)	To examine the relationships between triadic family interactions and maternal and parental OT and CORT.	Longitudinal	Coding of Triadic Interactions (gaze, affect, proximity position, and touch)	OT and CORT; Saliva and Plasma; ELISA	Paternal OT independently explained 22% of the variance in triadic synchrony. No relations between paternal CORT and triadic synchrony. The interaction between OT and CORT did not predict additional variance above and beyond the two hormones.
Gordon et al. (2010b)	*n* = 80 *n* = 62 at T2	T1 = 7.1 weeks (2.11) T2 = 24.8 weeks (4.38)	To understand the role of OT in the development of human parenting and its involvement in the transition to fatherhood.	Non-experimental prospective longitudinal	Coding of Parent–Infant Interaction (gaze, affect, vocalizations and touch)	OT; Plasma; ELISA	Paternal OT levels were associated with paternal stimulatory parenting behavior, but not with paternal affectionate parenting behavior.
Gordon et al. (2010c)	*n* = 43	T1: 2 months postpartum T2: 6 months postpartum	Examine the associations between PRL, OT, and paternal behaviors.	Longitudinal	Social Play-Coding of Parent–Infant Interaction (gaze, affect, vocalizations and touch) Toy Exploration—Coding of Parent–Infant Interaction (Parent gaze, Parent affect, Parent facilitation of infant toy exploration, Infant Toy Exploration, Paternal Coordinated Exploratory Play)	PRL determination: Chemiluminescent Microparticle Immunoassay (CMIA) technology; OT determination: ELISA	Averaged OT uniquely predicted Father–infant Affect Synchrony, while Averaged PRL was unrelated to Affect Synchrony. Averaged PRL uniquely predicted Coordinated Exploratory Play, but Averaged OT was unrelated to Coordinated Exploratory Play. In combination, PRL and OT explained 38 and 26% of the variance in Affect Synchrony and Coordinated Exploratory Play, respectively.
Feldman et al. (2010a)	*n* = 41	166.3 days (12.6)	To assess the involvement of the oxytocinergic system in human fathering and its consistency with parenting in other mammals; more specifically, to examine if fathers with high levels of stimulatory contact would show an increase in OT following a father–infant interaction.	Cross-sectional	Rating of father involvement on house-care and childcare responsibilities Coding of Parent–Infant Interaction (parental touch patterns and parent’s active engagement in exploratory)	OT; Saliva and Plasma; ELISA	Paternal stimulatory touch but not affectionate contact was related to plasma and salivary OT. OT increases in high stimulatory contact fathers but not in low stimulatory contact fathers, following parent–infant contact OT increased from the pre- to post-contact assessment among high stimulatory contact fathers.
Feldman et al. (2010b)	*n* = 19	157.1 days (11.9)	To examine: whether parental and infant OT, at both the baseline and post-interaction assessments, would be significantly correlated and if more sensitive parenting behavior would be associated with higher parental and infant OT.	Cross-sectional	Coding of Parent–Infant Interaction (parent/infant gaze, affect, vocalizations, touch)	OT; Saliva and Plasma; ELISA	OT increased in both parent and child following the contact interaction. Higher OT levels in parent and child were related to greater affect synchrony. Under conditions of high affect synchrony, infants with parents showing high OT had significantly higher OT than infants with parents with low OT.
Gettler et al. (2011)	*n* = 624 fathers and non-fathers	0–12 months	Clarify the role of T in human male reproductive strategy.	Observational longitudinal	Questionnaire (daily time spent providing physical care to children)	T; Saliva; EIA	Largest declines at waking (AM) and before bed (PM) T in new fathers. Fathers with newborns differed significantly for AM T compared with fathers with older infants. Fathers reporting higher involvement in childcare showed significantly lower values of AM and PM T compared with fathers reporting no care.
Feldman et al. (2011)	*n* = 41	166.3 days (12.6)	To assess the relation between OT and synchronous parent–infant interactions and to test the relation between OT and parent’s attachment relationships to infant.	Cross-sectional	Coding of Parent–Infant Interaction (parent/infant gaze, affect, vocalizations, touch) Yale Inventory of Parent Thought and Action Parenting Stress Index Questionnaire	OT; Saliva, Plasma and Urine; ELISA	Reported differences between the high- and low-Affect Synchrony groups were found for plasma and saliva OT. Plasma and saliva OT were correlated with parent Positive Engagement, Affect Synchrony, and Positive Communicative Sequences between parent and child. Plasma and saliva OT were associated with parents’ attachment relationships to infant and saliva OT was correlated with Parental Preoccupations.
Perini et al. (2012a, 2012b)	*n* = 37	T2: 2 months old	Examine if T levels during the transition to fatherhood are related to individual personality traits.	Longitudinal	N.A.	T determination: EIA	Lower repeated T levels in fathers compared to controls. Post hoc tests indicated that AUCg-T was significantly lower in fathers than in controls at T2. The changes in T levels did not reach statistical significance either within the group of fathers or within the group of controls.
Weisman et al. (2012)	*n* = 35	5 months (1.25)	To examine if OT administration to parent influences physiological and behavioral processes that support parental social engagement.	Double-blind, placebo-controlled, experimental	Positive and Negative Affect Schedule Coding of Parent–Infant Interaction (parent gaze, affect, touch and vocalizations; infant gaze, affect and exploratory play)	OT; Saliva; ELISA	Greater autonomic readiness for social engagement in the OT condition. Episodes of social reciprocity were longer in the OT condition. In the OT condition, fathers had longer episodes of touch (combined affectionate and stimulatory touch). Latencies to the first episode of father’s touch and social gaze at the infant were shorter in the OT condition.
Weisman et al. (2013a)	*n* = 35	5 months (1.25)	To test whether intranasal OT administration to the parent modulates father’s distance, motion characteristics and vocalization during interaction with his infant.	Double-blind, placebo-controlled, within-subject	Analysis of parent–infant motion (distance, speed and acceleration) Analysis of parent–infant speech turn-taking (father/infant vocalization; father/infant pause; silence, overlap ratio and synchrony ratio)	OT; Saliva; ELISA	OT modulates parental proximity to the infant, as well as the father’s head speed and head acceleration but not the father’s vocalization during dyadic interaction. Following OT administration, the maximum distance between father’s head and the infant was greater, and minimum distance was reached earlier, compared with the placebo condition.
Weisman et al. (2013b)	*n* = 35	5 months (1.25)	To investigate whether intranasal administration of OT to the parent has an effect on the parent’s and infant’s CORT response to a social stressor and to test if the degree of parent–infant synchrony moderates this effect.	Double-blind, placebo-controlled, within-subject	Coding of Parent–Infant Interaction (indices of dyadic exchange—social gaze and gaze synchrony) Parenting Stress Index	OT and CORT; Saliva; ELISA	CORT levels at the end of interaction (T4) were significantly lower than at T1, T2, and T3. Fathers’ CORT production was higher for the OT group compared to placebo. Fathers’ CORT was negatively correlated with the mean durations of father’s neutral affect; mean duration, frequency, total duration, and latency to father’s proprioceptive touch; frequency of gaze synchrony between father and infant; and touch mys-synchrony.
Weisman et al. (2014)	*n* = 35	4–8 months	Examine how OT administration change T levels and how T levels are related to parent–child social behaviors (gaze; parental touch; parental positive affect; parental vocalization; infant negative emotionality; infant object manipulation; infant negative vocalization).	Double-blind, placebo-controlled, within-subject	Coding of Parent–Infant Interaction (parental gaze, touch, affect and vocalization; infant gaze, negative emotionality, object manipulation and negative vocalization)	T and OT; Saliva; EIA	T was negatively correlated with the mean durations of parental vocalization and positively correlated with the latency to paternal vocalization. T correlated with frequency and proportion of father’s gaze to infant’s body. T marginally correlated with father’s self-reported weekly hours spent with infant.
							OT-induced change in T levels correlated with parent–child social behaviors; when controlling for basal T levels, the unique contribution of T-change in predicting behavior remains in most cases.
Gordon et al. (2017)	*n* = 80	T1: 1 month old T2: 6 months old	Examine how OT and T levels in new fathers across the first six months of parenthood shape parental behavior at 6 months.	Non-experimental, prospective longitudinal	Coding of Parent–Infant Interaction (parent gaze, vocalization, affect, touch and parent–infant proximity)	T; Plasma; Chemiluminescent Immunoassay (CLIA) technology OT; Plasma; ELISA	T at T1 negatively correlated with Parent–Infant Synchrony. T at T2 negatively correlated with Stimulatory Affectionate Touch. No correlations between OT and parental behavior at both time points. There is an interaction of T and OT in predicting parental behavior across time. Father–Infant Synchrony best predicted by hormones from T1 with a significant negative association to T; Paternal Affectionate. Touch is best predicted by hormones from T2, with a significant negative main effect for T and a significant interaction effect between OT and T. When T was low or medium, OT was uncorrelated to Affectionate Touch. When T was above average, OT was negatively correlated with Affectionate Touch.
							Stronger negative relation between T and quality of caregiving in fathers with lower CORT levels. Lower quality of postnatal caregiving associated with higher prenatal CORT levels.
Bos et al. (2018)	*n* = 56	6 weeks old	Examine the relation between observed quality of caregiving during parent–child interactions and pre- and postnatal T and CORT levels.	Longitudinal	Rating of Parent–Infant Interaction—quality of caregiving (sensitivity and cooperation)	T and CORT; Saliva; EIA	The quality of caregiving was unrelated to either postnatal T or CORT in the postnatal period. There was an interaction of quality of caregiving and T on CORT postnatally, caused by a stronger negative relation between CORT and quality of caregiving in fathers showing high T levels.
Kuo et al. (2018)	*n* = 298	Birth day—4 months	Examine how short-term hormonal changes during early dyadic interactions and fathers’ basal hormone levels predict their later involvement with their infants.	Observational longitudinal	Skin-to-skin contact with newborn (1 h). Childcare Activities Scale (percentage of involvement in infant care tasks)	T and CORT; Saliva; ELISA	Higher basal CORT at infant’s birth was related to greater involvement in direct care. Lower basal T at the second day after birth was related to greater involvement in direct care. Higher levels of basal CORT at the day of birth and at the second day were related to greater involvement in indirect care at follow-up. Lower levels of basal T on the second day were related to more involvement in indirect care. Fathers whose CORT increased more while holding their infants for the first time reported greater involvement in indirect care. Fathers with higher levels of basal CORT and increases in CORT while holding their infants for the first time, were more involved in play at 2–4 months postpartum.
Corpuz et al. (2020)	*n* = 220 (T2) *n* = 196 (T3)	T2: 3 months postpartum T3: 9–10 months postpartum	Examine how T levels are related to paternal care.	Longitudinal	The Laboratory Temperament. Assessment Battery Home Version (fear dimension). Coding Parent–Infant Interaction (Physical.	T determination: EIA	Although not significant, fathers with relatively accelerated T rebounds reported spending less time with their infants. Fathers who demonstrated accelerated T rebounds (from T2 to T3) exhibited significantly higher levels of paternal care quality.
					Affection, Warmth/Support, Listener Responsiveness, Humor, and Endearment) Time invested in parent–infant interaction (experience sampling method)		
Corpuz and Bugental (2020)	*n* = 220 (T2) *n* = 196 (T3)	T2: 3 months old T3: 9–10 months old	Examine individual differences in T levels after childbirth.	Longitudinal	N.A.	T determination: EIA	Death of a sibling/friend and the upheaval of one’s parental relationships each predict a younger age of sexual debut. Age of sexual debut did predict the rate of T recovery from T2 to T3: The younger one’s sexual debut, the more accelerated their T “rebound” from T2 and T3.
**Structural neuroimaging**							
Kim et al. (2014)	*n* = 16	2/4 weeks and 3–4 months	Investigate structural changes in fathers’ brains during the first 4 months postpartum.	Observational, longitudinal	Coding of Parent–Infant Interaction (Paternal sensitivity and intrusiveness)	GMV whole-brain Voxel-based morphometry; correlations between GMV and parenting behaviors	Decreased GMV in the OFC was associated with increased intrusive parenting behaviors.
**Functional neuroimaging**							
Atzil et al. (2012)	*n* = 15	4–6 months	Examine synchrony in mothers’ and fathers’ brain responses to own-infant cues.	Exploratory	N.A.	Whole-brain and ROI-based; contrasts; correlations between hormones and brain areas activation	Increased activations in cognitive areas related with decreased OT levels.
Kuo et al. (2012)	*n* = 10	2–4 months	Investigate neural responses to infants in fathers and the association between neural activity, T, and parental behavior.	Observational, cross-sectional	Coding of Parent–Infant Interaction (Parental Sensitivity and Parental Reciprocity)	Whole-brain and ROI-based; contrasts; correlations between ROI activation, parenting and hormonal measures	Increased activity in emotion regulation and mentalizing circuits in response to own infant. Increased activity in salience, reflexive caring, emotion regulation, and mentalizing circuits in response to baby (vs. doll). Decreased activation in right orbitofrontal cortex in response to own infant was associated with greater paternal sensitivity and reciprocity. Increased T levels after interaction with own infant were associated with increased activation in the left caudate in response to own infant.
Abraham et al. (2014)	*n =* 21	11 (6.67) months	Investigate parental brain responses to infant stimuli.	Observational	Coding of Parent–Infant Interaction (Parent–Infant Synchrony)	ROI-based contrasts and connectivity; correlations between brain activation, parenting and hormones; mediation analysis.	Increased activation in socio-cognitive and mentalizing networks was associated with OT levels and parent–infant synchrony.
Kim et al. (2015)	*n* = 19	T1 = 1 month T2 = 3/4 months	Investigate associations between parental thoughts/actions and neural activation in fathers in the neonatal period with infant outcomes.	Prospective longitudinal	Yale Inventory of Parental Thoughts and Actions—Revised (YIPTA-R)	Whole-brain; contrasts; correlation of neural responses with positive parenting	Increased activity in the auditory cortex, thalamus/hypothalamus and caudate in response to own infant baby cries at the first month postpartum were associated with positive parenting in fathers.
Abraham et al. (2018)	*n =* 21	11 (6.67) months	Investigate the neurobiological basis of parental empathy.	Longitudinal	N.A.	ROI; connectivity	Increased connectivity within and between two empathy networks (embodied simulation and mentalizing) when viewing own infant videos.
Li et al. (2018)	*n =* 39	<4 months	Investigate variation in paternal neural responses to infant crying.	Observational	Parenting Stress Index—Short Form	Both whole-brain and ROI-based; contrasts; correlations between paternal characteristics and neural responses.	Own and unknown infant cries activate empathy and approach motivation circuits. Decreased dorsal anterior cingulate cortex and anterior insula activity in response to infant cries in older fathers. Increased neural responses were associated with lower infant age. Increased activation of right posterior temporal gyrus in fathers who reported negative emotions in response to own infant’s cry.
Paternina-Die et al. (2020)	*n* = 20	70.25 (49.21) days	Investigate changes in cortical volume, thickness, and area in fatherhood and their association with indicators of paternal experience.	Longitudinal	N.A.	Whole-brain (cortical regions); contrasts.	Increased activity in default mode and dorsal attention networks in response to own baby (vs. unknown baby).

SNPs, Single nucleotide polymorphisms; OT, Oxytocin; PRL, Prolactin; CORT, Cortisol; T, Testosterone; GMV, Gray matter volume; OFC, Orbitofrontal cortex; and ROI, Region-of-interest.

#### Endocrine and Molecular Biomarkers

In the endocrine and molecular domains, 21 articles met the inclusion criteria (*cf*. Table 1), of which 19 focused on endocrine changes and two combined both endocrine and SNPs analysis. Within the endocrine studies, six studies focused exclusively on Oxytocin (OT; Feldman et al., 2010a,b, 2011; Gordon et al., 2010b; Weisman et al., 2012, 2013a), five studies focused on Testosterone (T; Gettler et al., 2011; Perini et al., 2012a,b; Corpuz and Bugental, 2020; Corpuz et al., 2020), one study focused on Prolactin (PRL; Delahunty et al., 2007), two studies analyzed both OT and T (Weisman et al., 2014; Gordon et al., 2017), another two studies observed OT and Cortisol (CORT; Gordon et al., 2010a; Weisman et al., 2013b), one OT and PRL (Gordon et al., 2010c), and two T and CORT (Bos et al., 2018; Kuo et al., 2018). Regarding molecular studies, two studies analyzed both OXTR and CD38 genetic polymorphisms (Feldman et al., 2012, 2013).

The majority of studies reported longitudinal quantitative data (*n* = 13, Delahunty et al., 2007; Gordon et al., 2010a,b,c, 2017; Gettler et al., 2011; Perini et al., 2012a,b; Feldman et al., 2013; Bos et al., 2018; Kuo et al., 2018; Corpuz and Bugental, 2020; Corpuz et al., 2020). Seven of which reported data from pre-birth, but we only considered results within the 0–12 months postpartum period, meeting the criterion for inclusion. Eight studies reported on cross-sectional quantitative data (Feldman et al., 2010a,b, 2011, 2012; Weisman et al., 2012, 2013a,b, 2014). Samples ranged from 35 to 465 participants, and the father’s mean age was 29.33 years. In 10 of these 21 articles, infants were the first child for the father. There was variation regarding the assessment of parenting outcomes (subjective parental care quality and parental behaviors reported) associated with endocrinal and molecular biomarkers (*cf*. Table 1).

Besides controlling for the effect of time of day for hormone values, the majority of the reviewed studies included the following covariates in their models or examined potential correlations with those prior to data analysis: parent age (Feldman et al., 2010a,b, 2011, 2012, 2013; Gordon et al., 2010b,c; Perini et al., 2012a,b; Weisman et al., 2014; Bos et al., 2018; Kuo et al., 2018; Corpuz and Bugental, 2020); education (Feldman et al., 2010a, 2012, 2013; Gordon et al., 2010c; Bos et al., 2018); height (Feldman et al., 2010b, 2011, 2012, 2013; Gordon et al., 2010b,c); weight or body mass index (Feldman et al., 2010a,b, 2011, 2012, 2013; Gordon et al., 2010b,c; Perini et al., 2012a,b; Corpuz and Bugental, 2020); smoking (Feldman et al., 2010a,b, 2011, 2012, 2013; Gordon et al., 2010b,c); time of last meal (Feldman et al., 2010b, 2011, 2012, 2013; Gordon et al., 2010b,c); use of medication (Feldman et al., 2010b, 2011; Gordon et al., 2010b,c); and number of children/parity (Gettler et al., 2011; Bos et al., 2018; Kuo et al., 2018). Anecdotally, other studies observed variables related to parents such as: gender (Feldman et al., 2012), religiosity (Feldman et al., 2010a), ethnicity (Corpuz and Bugental, 2020), birth order (Feldman et al., 2010a), hours of employment (Feldman et al., 2010a), parental status (Feldman et al., 2012), parental anxiety (Gordon et al., 2010c), parenting stress (Gordon et al., 2010c; Weisman et al., 2013b), and psychosocial stress (Gettler et al., 2011), sleep quality, duration or disruption (Gettler et al., 2011; Perini et al., 2012b). Others observed variables related to the infant, such as infant’s sex (Kuo et al., 2018) and age (Gordon et al., 2010c). Similarly, a couple of studies observed variables related to the parental marital relationship, such as: relationship duration (Perini et al., 2012a,b), time spent together as a couple and with significant others (Perini et al., 2012b), marital status (Kuo et al., 2018); and finally, a few other variables were considered across studies, such as consumption of caffeine (Perini et al., 2012a,b), activities during the day of saliva sampling (e.g., time with partner and baby; Perini et al., 2012b); hormone levels (Weisman et al., 2014); drug order (i.e., OT first vs. placebo first; Weisman et al., 2014); and maternal care (Corpuz et al., 2020).

##### Genetic Studies

To our knowledge, only two studies addressed the association between genetic variability in two different genes (CD38 and OXTR) and paternal behavior and endocrine changes. Specifically, Feldman et al. (2012, 2013) showed both fathers and non-fathers carriers of the C allele in a particular SNP (rs3796863) of the CD38 gene produced lower plasma OT levels. The same profile was observed for carriers of the GG (rs2254298) and TT (rs1042778) genotypes of the OXTR gene. Additionally, fathers with CD38 CC genotype showed smaller frequency in touch toward their children, compared to those carrying the AA/AC genotype, and fathers with OXTR rs1042778 TT genotype also provided less touch to their infant. Furthermore, among fathers, longer parent–infant gaze synchrony was predicted by the combination of CD38 low-risk A allele and high plasma OT.

##### Endocrine Studies

*Oxytocin*. Only one study compared OT levels between fathers and non-fathers, showing that parents had higher OT levels than non-parents (Feldman et al., 2012). Father’s OT hormone basal levels differ in terms of tissue used for OT collection, with a range of 306.01–405.10 pg/ml for plasma (Feldman et al., 2010b, 2011; Gordon et al., 2010a,b) and 7.09–23.20 pg/ml for saliva samples (Feldman et al., 2010a, 2011; Weisman et al., 2012), with results showing that plasma and saliva OT levels are interrelated (Feldman et al., 2010a, 2011). One study reported values for basal OT urine samples (*M* = 9.81 pg/ml, SEM = 2.03), which does not correlate with both saliva and plasma OT levels (Feldman et al., 2011). There is a time effect in OT levels in fathers, in that its values rise from the first week postpartum up to 6 months postpartum (Gordon et al., 2010b). Additionally, OT levels show high intraindividual stability across time in fathers (Feldman et al., 2010a, 2011, 2013; Gordon et al., 2010b; Weisman et al., 2012) and basal levels of this hormone increase after interactions with own infants (Feldman et al., 2010a, 2011; Gordon et al., 2010b; Weisman et al., 2012).

Furthermore, higher OT levels in fathers correlate with increased time spent in father-to-infant touch (Feldman et al., 2012), related with stimulatory contact (proprioceptive contact, tactile stimulation, and object presentation), but not with affectionate touch (Gordon et al., 2010b). Moreover, fathers with high levels of stimulatory contact show an increase in OT after parent–infant interaction compared to fathers with low levels of stimulatory contact (Feldman et al., 2010a). Feldman et al. (2011) showed that both plasma and salivary OT correlated with positive engagement, affect synchrony, and positive communicative sequences between parent and child. Higher OT levels in fathers were also associated with early parental care (Feldman et al., 2013). Furthermore, in a mother–father–infant interaction, Gordon et al. (2010a) showed that father’s OT independently predicted triadic synchrony, suggesting that greater OT levels in fathers contribute to increased synchrony in triadic interactions.

In an OT administration paradigm, Weisman et al. (2012) showed that fathers in the OT condition revealed higher respiratory sinus arrhythmia (a measure of the parasympathetic activity that relates to orientation, attention, and social engagement; Porges, 1995), suggesting that increased OT levels are associated with higher fitness for social engagement between father and infant. Furthermore, fathers in the OT condition showed more infant-oriented positive vocalizations, higher father’s social reciprocity, encouragement of infant orientation to the social context, and extended periods of touch. On the contrary, Weisman et al. (2013a) showed that OT administration seems to have no effect on vocalizations toward the child, but instead was associated with increased father’s head motion (head speed and acceleration during dyadic interaction), and shorter distance toward the child, reflecting a more pronounced infant-directed repertoire, which contributes to the affiliative process.

Moreover, no correlation was found between OT and father’s state or trait anxiety or stress in parenting role (Gordon et al., 2010c).

*Prolactin*. Two studies analyzed changes in PRL in fathers during the first 6 months postpartum (Delahunty et al., 2007; Gordon et al., 2010c). PRL baseline plasma levels differed in each study, with average values ranging from 10.6 ng/ml (Delahunty et al., 2007) to 317.356 ng/ml (Gordon et al., 2010c). In an experimental setting, no differences emerged in PRL levels between non-fathers holding a doll and fathers holding their infants in the early postpartum period (first 2–3 weeks after birth; Delahunty et al., 2007). However, significant differences emerged with the second child of these same fathers, reflected in a greater increase in PRL levels after holding their second babies compared to the PRL levels registered after holding their first child (Delahunty et al., 2007). In line with this result, a non-significant trend emerged showing that men who have above average contact with children have a greater percentage increase in PRL, compared to men with less contact with children (i.e., considering the estimated percentage of time over their lifetimes that had spent with infants and how many younger siblings did they have; Delahunty et al., 2007). Furthermore, the quantity of time spent with one’s own infant seems to influence PRL levels. Fathers who spent all day at home with their infants had smaller PRL increases compared to fathers who had spent less than 3 h with their newborn at the 2–3 weeks postpartum period, and no differences in baseline PRL levels were found between groups (Delahunty et al., 2007). On the contrary, at 2 months postpartum, fathers having less time holding their babies showed higher baseline PRL levels compared to men who held their babies for longer periods, and no differences were found in percentage change of PRL levels (Delahunty et al., 2007). In Gordon et al. (2010c), PRL levels were stable over time (at 2 and 6 months postpartum) and higher PRL average scores correlated and significantly predicted father’s coordinated exploratory play with own infant. Average PRL also correlated marginally with affect synchrony between father and infant in a social play interaction (Gordon et al., 2010c).

*Testosterone*. Apart from one study, which used plasma as the tissue for T determination (Gordon et al., 2017), all other studies collected saliva samples. Perini et al. (2012b) compared fathers at 2 months postpartum with no-fathers as controls, reporting that T was significantly lower in fathers. Longitudinal quantitative data revealed paternal T rebounds across the postpartum period (Gordon et al., 2017; Corpuz and Bugental, 2020; Corpuz et al., 2020). This recovery is observed already at the postpartum day two, where paternal T was significantly higher (*M* = 94.39 pg/ml, *SE* = 2.57 pg/ml) when compared to the infant’s birth day (*M* = 82.18 pg/ml, *SE* = 2.48 pg/ml; Kuo et al., 2018). Four studies explored the differences in paternal T while taking into account father–infant interaction. Fathers who reported spending more time in childcare activities revealed significantly lower T levels compared to fathers with no involvement (Gettler et al., 2011). Fathers with lower T on their children’s second day of life reported greater involvement in direct (e.g., bathing) and indirect (e.g., washing infant’s clothes) childcare at approximately 16 weeks after postpartum (Kuo et al., 2018). Although it did not reach statistical significance, fathers with relatively accelerated T rebounds (i.e., higher increase of T levels after the descent of male T from pre-birth to the postpartum period and first year of infant’s life) reported spending less time with their infants (Corpuz et al., 2020). Regarding the quality of care, Bos et al. (2018) reported that at 6 weeks postpartum parental sensitivity and cooperation were unrelated to paternal T. In contrast, higher quality of paternal care was reported by fathers with accelerated T rebound (Corpuz et al., 2020). This result might be unexpected taking into account the previous findings on decreased time spent with infants associated with accelerated T rebounds, as well as the results found in two other studies, pointing to negative correlations between paternal T and fathers’ affectionate touch (Weisman et al., 2014; Gordon et al., 2017). Gordon et al. (2017) also reported a negative correlation between paternal T levels and parent–infant behavioral synchrony at 1 month postpartum, even though this correlation did not emerge at 6 months postpartum.

*Cortisol*. Studies show that on the infant’s birth day, father’s basal CORT levels measured immediately before holding their newborns for the first time decline significantly after, and rebound close to the mentioned father’s basal CORT baseline levels (*M* = −2.23 μg/dl, *SD* = 0.80) on day two postpartum (Kuo et al., 2018). As reported in Gordon et al. (2010a), CORT levels are described to be higher at two (5.38 pg/ml, *SD* = 2.14) and 6 months post-partum (5.60 pg/ml, *SD* = 1.39). Furthermore, a greater father’s involvement in play and childcare at 2–4 months postpartum was associated with father’s greater basal CORT levels and reactivity to first time holding their babies at infant’s birth day (Kuo et al., 2018). Also, father’s CORT levels significantly drop after parent–infant interaction across the first 4–8 months postpartum (Weisman et al., 2013b). At 4–8 months postpartum period, father’s CORT basal level (pre-parent–infant interaction) negatively correlated with father’s proprioceptive touch (mean duration, frequency, proportion, and latency) toward the child; with father’s neutral affect toward own infant; with frequency of gaze synchrony between father and child and with touch mys-synchrony (i.e., father’s proprioceptive touch toward the child coinciding with infant’s avert gazing from father; Weisman et al., 2013b).

##### Interactions Between Different Hormones

Six studies analyzing more than one hormone and possible interactions between them are described in this section.

*Oxytocin and Cortisol*. Relative to OT and CORT, the results are somewhat contradictory. In Weisman et al. (2013b), fathers who had self-administered OT showed an increased CORT response to a stress paradigm (face-to-face still-face paradigm). Nevertheless, in Gordon et al. (2010a), father’s plasma OT and salivary CORT were not correlated at both 2 months and 6 months postpartum, and OT and CORT interaction did not predict additional variance over and above the two hormones relative to synchrony in a triadic interaction.

*Oxytocin and Prolactin*. In the 2 months postpartum period, no association between PRL and OT was found in the Gordon et al. (2010c) study. However, by the sixth month postpartum, the authors found a positive association between the two hormones. Furthermore, in combination, PRL and OT explained 38 and 26% of the variance in Affect Synchrony and Coordinated Exploratory Play, respectively, in a father–infant interaction paradigm.

*Oxytocin and Testosterone*. One study (Gordon et al., 2017) reported no association between paternal T and OT *per se*. Nevertheless, the authors explored the interaction of T and OT in predicting parental behavior. At 6 months postpartum, when T was low or medium, OT was uncorrelated to paternal affectionate touch. However, when T was above average, OT was negatively correlated with paternal affectionate touch.

When OT was self-administered (Weisman et al., 2014), fathers at 4–8 months postpartum revealed higher T levels, compared to the placebo condition. The OT-induced change in paternal T levels was correlated with positive affect, social gaze, touch, and vocal synchrony observed during a father–infant interaction conducted 45 min after OT administration. The unique contribution of T-change remained in most cases even when controlling for basal T levels.

*Cortisol and Testosterone*. Bos et al. (2018) found a negative relation between CORT and quality of caregiving (i.e., parental sensitivity and cooperation) in fathers with higher T, at 6 weeks postpartum.

#### Structural Neuroimaging Research

Only one longitudinal study used structural MRI data to assess brain changes during the postpartum period (Kim et al., 2014), examining gray matter volume (GMV) data (voxel-based morphometry) in 16 fathers, assessed at two timepoints (2–4 weeks and 12–16 weeks postpartum). While controlling for father’s age, parity (primiparous or multiparous status), and scan intervals, the study showed that the decrease in the OFC volume was associated with higher paternal intrusiveness levels, especially with forcing behaviors (i.e., the physical manipulation of the infant’s body).

#### Functional Neuroimaging Research

Seven studies included functional neuroimaging methods. Of those, three (43%) followed a longitudinal design (Kim et al., 2015; Abraham et al., 2018; Paternina-Die et al., 2020; for the purpose of this review, only data collected within the 0–12 months postpartum period were included) and four (57%) followed a cross-sectional design (Atzil et al., 2012; Kuo et al., 2012; Abraham et al., 2014; Li et al., 2018). Samples ranged from 10 to 48 participants, with father’s mean age ranging from 29.3 to 37.68 years. Overall, studies included both first-time and veteran parents. Regarding the inclusion of covariates (either prior to the data analysis or in the models), besides controlling for diurnal variability in hormones when applicable, studies considered the father’s age (Paternina-Die et al., 2020), hormone plasma levels (Atzil et al., 2012), head motion parameters (Kuo et al., 2012; Paternina-Die et al., 2020), parity (Kim et al., 2015), paternal and infant characteristics (Li et al., 2018) and principal components for cry ratings (Li et al., 2018).

Infant stimuli were used across studies to evaluate fMRI responses, with two studies using auditory stimuli (baby-cry; Kim et al., 2015; Li et al., 2018) and five using visual stimuli, either pictures of the infant (Paternina-Die et al., 2020), videos of parent–infant interactions (Abraham et al., 2014, 2018) or infants’ emotional faces (Atzil et al., 2012; Kuo et al., 2012).

##### Neural Activity—BOLD Response

Neural responses to diverse infant stimuli have been investigated in five studies. The study by Li et al. (2018) reports brain function of first-time fathers in response to their own infant cry, a standardized unknown infant cry, and an auditory control stimulus. In this study, fathers were also asked to subjectively rate each infant cry stimulus, as annoyed, distressed, upset, angry, irritated, and aversive. The authors found widespread activations in response to both own and unknown infant cries in regions underlying empathy and approach motivation networks. Specifically, activations were observed in the medial prefrontal cortex, the bilateral anterior insula and the inferior frontal gyrus, the bilateral striatum, bilateral thalamus, bilateral auditory cortex, bilateral posterior cingulate, and bilateral midbrain. Additionally, the authors found that infant age, paternal age, and paternal emotional reactions influenced the neural responses of fathers to infant cries. Specifically, results showed that fathers who rated the unknown infant cry as more aversive exhibited greater neural activation in auditory cortices and decreased activation in motivation networks (thalamus and left caudate). In relation to parent characteristics, the authors also found that paternal age was negatively correlated with the neural response to own infant cries in both the dorsal anterior cingulate cortex and the right postcentral gyrus. Finally, younger infant age was found to be associated with increased neural responses in fathers.

The study by Kim et al. (2015) also assessed the neural responses of fathers to own and unknown infant cry sounds. Results showed that positive parenting (i.e., the positive experiences of parenting) was positively associated with brain activations in the right middle temporal gyrus (the auditory cortex), the thalamus, the hypothalamus, and the left caudate.

Abraham et al. (2014) measured parental brain responses to infant-related cues in secondary caregiving fathers, defining these as the father raising the infant within heterosexual relationships. Parent–infant interactions at the homebound were videotaped and used as stimuli during the fMRI scanner session, and the following contrasts were observed: Self–Infant Interaction > Self, and Self–Infant Interaction > Unfamiliar Parent–Infant Interaction. Passive viewing of the videotapes elicited activations in areas related to emotional processing and mentalizing networks: the bilateral amygdala, the ventral anterior cingulate cortex, the left inferior frontal gyrus/insular cortex, the ventral tegmental area, the bilateral superior temporal sulcus, the ventromedial prefrontal cortex, the temporal poles, and the lateral frontopolar cortex. Moreover, Abraham et al. (2014) observed that parent–infant synchrony was correlated with superior temporal sulcus activation in secondary caregiving fathers.

Another study reported on the neural responses of fathers to videos of their own neutral or smiling infant (OWN), other unknown matched infant (OTHER), as well as control stimuli (DOLL; Kuo et al., 2012). Whole-brain analysis for OWN > OTHER contrasts showed increased activity in regions underlying emotion regulation (bilateral inferior frontal gyrus) and empathic/mentalizing networks [bilateral supramarginal (parietal) gyrus and bilateral middle temporal gyrus]. Region-of-interest analysis for OWN > OTHER showed greater activations to own baby in the right superior frontal gyrus, the right inferior frontal gyrus, the right caudate, the left caudate, and the right orbitofrontal cortex. In relation to the contrast of baby (average of both own and other infant) vs. DOLL, greater activation was reported in fathers in the sensory/salience, reflexive caring, emotion regulation, and empathic/mentalizing networks, namely in the bilateral caudate, the orbitofrontal cortex, the superior frontal gyrus, the bilateral middle temporal lobes, the bilateral superior parietal lobules and a network of lateral frontal regions. Kuo et al. (2012) additionally found that the activation in the right orbitofrontal cortex was negatively correlated with parental sensitivity (i.e., the parent’s ability to perceive the infant’s signals and to respond adequately to the infant, allowing the latter to determine the process of the interaction) and parental reciprocity (i.e., reciprocal interactions between parent and infant).

The whole-brain study by Atzil et al. (2012), used similar stimuli and found that fathers showed greater activation in socio-cognitive regions (e.g., left medial prefrontal cortex and left precuneus) in response to their infant (OWN infant > standard infant contrast).

Finally, in the whole-brain study by Paternina-Die et al. (2020), neural responses of first-time fathers were assessed using pictures of their own and unknown infants’ faces. Regarding the OWN > OTHER contrast, activations were found in brain regions that overlap with the default mode and the dorsal attention networks, including bilateral precuneus, middle temporal gyrus, posterior cingulate cortex, middle occipital lobes, and right-sided activations in the inferior frontal/parietal gyrus, the angular/pre-central gyrus, the inferior/posterior temporal gyrus, and the superior occipital lobe.

##### Functional Connectivity

The study by Abraham et al. (2014) analyzed functional connectivity between the amygdala and the superior temporal sulcus in fathers which was found to be correlated with the time spent in direct infant care.

Abraham et al. (2018) examined the connectivity in three brain networks of interest, among secondary caregiving fathers, in response to neutral natural interactions and attachment-related video vignettes (fathers were instructed to interact freely with their own infant—Self–Infant Interaction) and found increased connectivity within and between embodied simulation and mentalizing networks in fathers when viewing their interactions with their own infant (vs. unknown infant interactions).

##### Interaction Between Endocrine and Neural Correlates

Two studies observed associations between the hormone OT and neural responses. The study by Abraham et al. (2014) suggested that the neural activity of the superior temporal sulcus was correlated with OT levels in secondary caregiving fathers and the father–infant synchrony was indirectly affected by the superior temporal sulcus, through increases in OT. Atzil et al. (2012) observed that decreases in OT were associated with higher activations in cognitive areas (e.g., dorsolateral prefrontal cortex, dorsal anterior cingulate cortex, and pre-central gyrus) in response to own-infant videos. Finally, T levels following father–infant interactions were positively correlated with the left caudate activation in response to own infant videos in the Kuo et al. (2012) study.

## Discussion

In our scoping review, we identified 29 primary studies published between 2005 and 2020 addressing neurobiological correlates of fatherhood. Specifically, 19 studies were retrieved for neuroendocrine biomarkers, two for SNPs of candidate genes, one for structural neuroimaging, and seven for brain activity or functional connectivity. Overall, there seems to exist some evidence in favor of significant neuroendocrine and neurofunctional changes, as well as a role for genetic variability in the transition to parenthood in fathers. On the contrary, there was limited or non-existent research focusing on structural brain changes and other molecular mechanisms such as epigenetic modifications associated with fatherhood outcomes (e.g., the quality of subjective paternal care and paternal behaviors reported) during the postpartum period.

Regarding the neuroendocrine changes and genetic variability, we found 19 studies focusing on hormonal alterations in fathers and two studies focusing on polymorphic variations on candidate genes that influence paternal behavior. Paternal caregiving behaviors in human fathers toward their infants were shown to be associated with paternal hormonal levels, such as OT, CORT, T, and PRL. Findings suggest that positive engagement, father–child touch, stimulating contact, parental care, affect synchrony, and positive communicative sequences are related to higher OT levels (Feldman et al., 2010b, 2011, 2012, 2013). Furthermore, studies where OT was experimentally increased *via* nasal administration also show that this hormone increases paternal caregiving behaviors such as touch, social reciprocity, greater physical proximity to the child, and readiness for social engagement (Weisman et al., 2012, 2013a).

This evidence emphasized how OT is relevant for paternal behavior toward their children, corroborating the vast literature within the scope of maternal behavior which consistently demonstrated that OT correlates with mother–infant attachment and interaction quality (Feldman et al., 2007). Moreover, OT can also affect paternal behavior indirectly by altering other hormones’ levels. Indeed, OT administration increases CORT (Weisman et al., 2013b) and T levels (Weisman et al., 2014). These OT-induced changes in CORT were associated with lower paternal social interaction behavior with their babies, which may suggest an important integration of both stress and bonding neuro-endocrine systems in the transition to parenthood. Additionally, OT-induced changes in T correlated with greater paternal behavior, including positive arousal, social gaze, and vocal synchrony. These results go in line with Weisman and Feldman (2013), who suggest that OT manipulation, especially the one with T levels, increases reward and social salience in the context of social interactions through interactions with the dopamine mesolimbic pathway (Hermans et al., 2010), possibly facilitating father’s caregiving commitment.

In fact, OT and T do not operate independently, but instead they interact with each other which collectively may influence parenting behavior. An Estrogen Response Element was already identified in the human OT gene and estrogens were found to upregulate oxytocin production in specific subpopulations of OT neurons (Richard and Zingg, 1990). More recent findings also point out the possibility of a direct regulation of androgens on the OT gene, through the evidence of a co-localization of androgen receptors (AR), in OT neurons of the paraventricular nucleus of the hypothalamus (Dai et al., 2017). Additionally, T is known to be metabolized to estradiol, which in turn increases the synthesis of OT (Cornil et al., 2006), a mechanism hypothesized to mediate maternal behavior. In this regard, an fMRI study with young women revealed increased activation of the limbic-related structures after T administration in response to infant crying when compared to a control sound, shedding light on the neuroendocrine regulation of maternal care (Bos et al., 2010). Regarding fathers, a study included in the current review also calls attention to this interplay, showing that intranasal OT administration, compared to placebo, leads to a short-term alteration in T levels among fathers while socially interacting with their infants (Weisman et al., 2014). Taken together, this evidence highlights a complex hormonal control of parenting behavior and urges future studies to explore this interplay to provide a more comprehensive understanding of the mechanisms underlying parenting behavior.

Despite the above-reported evidence about the interplay between OT and T, the studies looking at the individual role of T and parenting behavior yield some inconsistent results. Whereas some studies reported that lower T levels are related to greater father involvement in childcare (Gettler et al., 2011; Weisman et al., 2014; Gordon et al., 2017; Kuo et al., 2018), others report no relationship between these two variables (Bos et al., 2018) or even contrasting results (Corpuz et al., 2020). One possible caveat accounting for some of the results’ discrepancies could be the fact that the measurements and behaviors analyzed are different among studies, ranging from questionnaires to father–infant interaction paradigms. Furthermore, a common feature of most of these studies is that they assessed the relationship between T levels and father’s involvement with their first child. The evaluation of whether the number of children influences father’s involvement in childcare and whether this is related to higher or lower T levels should be tackled by future studies. Nevertheless, it should be acknowledged that the relationship between T and paternal behavior is not unidimensional. Parenting involves not only nurturant but also competitive phenomena (e.g., infant defense), which could be translated in different T levels (van Anders, 2013). Through partitioning intimacy and aggression, the Steroid/Peptide Theory of Social Bonds (van Anders et al., 2011) hypothesized that low T is only linked to parental contexts perceived as nurturant, while high T is linked to the invocation of defense or protection in the parental context (e.g., infant crying). Finally, cultural norms can also count on the paternal T variation, namely in terms of the degree of involvement with direct care (Gettler, 2014).

Regarding PRL, we found two conflicting studies, whereas in Gordon et al. (2010c) higher PRL levels seem to be related to greater involvement of the father in play moments with the child, Delahunty et al. (2007) found that lower PRL levels were related with increased time spent with the child. In fact, literature has been showing that higher levels of PRL in human males after childbirth seem to be associated with higher paternal behaviors, by decreasing parents’ libido, contributing to a shift from reproductive to nurturing behavior (Hashemian et al., 2016). Nevertheless, the biological role of PRL in human fathers is still under investigation and so novel studies are needed in order to clarify this association.

With regards to CORT, levels of this hormone tend to remain stable throughout the postpartum period (Gordon et al., 2010a). However, some authors suggest that higher basal CORT levels are related to greater paternal involvement in caregiving between two and 4 months postpartum (Kuo et al., 2018) and this pattern seems to take the opposite direction between four and 8 months postpartum in what concerns measures of father–infant interaction (i.e., touch frequency, gaze synchrony, and social gaze; Weisman et al., 2013b). Future studies should be able to disentangle the contribution of CORT levels to fatherhood by observing the associations between CORT levels and each dimension of interest (measures of caregiving vs. measures of father–infant interaction) across time.

Including neurogenetic studies in addition to peripheral measures is an important strategy in understanding the role of OT in parenthood. Polymorphisms in genes such as the OXTR or CD38 that encode elements of oxytocinergic brain pathways are shown to be plausible key regulators of paternal behavior and investment. As described in the present review, carriers of the CC genotype of the CD38 gene, as well as OXTR rs1042778 TT genotype carriers, showed to be fathers displaying low touch frequency toward their children. Empirical studies with mothers show that OXTR SNPs also play a role in maternal behavior, including more sensitive and more positive parenting (Bakermans-Kranenburg and Van Ijzendoorn, 2008; Michalska et al., 2014), which reinforces the importance to look for such dimension also among fathers. Furthermore, there is evidence showing how SNPs on the OXTR associate with structural brain changes, including greater gray matter and smaller volumes of both left and right amygdala (Furman et al., 2011). Taken together, these findings suggest that these genetic variations may contribute to individual variability in paternal behavior by altering OT release patterns.

Surprisingly, we did not find any evidence for epigenetic mechanisms related to fatherhood which we consider to be a relevant gap in the literature given the fact that some of the existing studies do point to the influence of these mechanisms in maternal behavior. A recent study from Toepfer et al. (2019) points to dynamic DNAm changes in the maternal OT gene during pregnancy, which predicts postpartum maternal intrusiveness. Additionally, Feldman et al. (2012) showed that parents who reported greater parental care received from their own parents had higher plasma OT levels and showed more behaviors of touch toward their infants. These data may be the result of how fathers’ environmental factors during their own neurodevelopmental trajectories are embedded in molecular mechanisms such as epigenetics modifications, contributing to higher/lower gene expression and consequently influencing hormonal response and parental behavior. Indeed, akin to the mother–daughter transmission of maternal care and behavior seen in mammals (Champagne, 2008), paternal care can also impact biological systems underneath stress reactivity and social behavior in offspring. This, in turn, can reflect meiotic and transgenerational epigenetic mechanisms associated with paternal behavior patterns, requiring further investigation. In fact, animal research is considerably advanced and refined in terms of mechanistic details on how paternal germ cells imbeds information from the environment and further passes it to future generations, making them more vulnerable or resilient to stress and/or psychopathology (for a review, see Cunningham et al., 2021). Given the lack of literature and work done with human fathers regarding how epigenetic mechanisms influence paternal caregiving, rodent work should be considered not only as an essential theoretical background for future work in human fathers, but also as a crucial source to inform and interpret future human studies results. Indeed, rodent work in this area is very much advanced and has isolated physiological mechanisms related to epigenetic that will be difficult to replicate in humans. Focus on animal research should then never be ignored in future human studies.

Regarding brain structure changes associated with fatherhood, we only found one study (Kim et al., 2014), highlighting the scarcity of research on specific postpartum brain changes in fathers in the postpartum period. The included study is distinguished by its longitudinal design showing that GMV decrease in the right orbitofrontal cortex was associated with paternal intrusiveness, confirming that structural brain plasticity is associated with caregiving processes. Overall, these structural alterations may constitute a mechanism for the functional adaptations observed in fathers during the postpartum period, increasing parenthood-related repertoires (e.g., for parental motivation; resource allocation). More longitudinal studies that investigate the association between neurostructural changes and fatherhood repertoire in the postpartum period are deemed necessary to further progress in the field.

Brain activity using fMRI in the one-year postpartum period in response to an infant’s auditory or visual stimuli was observed by five studies. Underlying the expression of parental behavior, activity in several networks that share common brain regions and that can be coactivated was reported. Namely, empathy and approach motivation (Li et al., 2018), emotional processing and mentalizing (Atzil et al., 2012; Kuo et al., 2012; Abraham et al., 2014), emotion regulation (Kuo et al., 2012), and dorsal attention and default mode networks (Paternina-Die et al., 2020). Brain activity findings seem to be in accordance with those arising from structural neuroimaging (Kim et al., 2014). For example, right orbitofrontal cortex activation was associated with lower paternal sensitivity and reciprocity (Kuo et al., 2012) and gray matter volume increases in the same brain region with parental intrusiveness (Kim et al., 2014). Also, the caudate was activated while watching video clips of own (vs. control) infants (Kuo et al., 2012), and the caudate GMV was found to be increased during the postpartum period (Kim et al., 2014) which may well be the result of increased synaptogenesis and neurogenesis mechanisms associated to the acquisition of new caregiving skills and parental behaviors in the transition to fatherhood. Further studies observing the association between structural and functional brain changes and its effect on parenthood in fathers would progress the field.

The methodology applied to fMRI data was relatively consistent throughout the literature, with comprehensive descriptions of the contrasts carried out and the image analysis methods. The use of whole-brain and ROI approaches was balanced, with the latter generally justified across studies. However, caution should be warranted to the use of different statistical thresholds, as well as the correction for multiple comparisons across studies. Also, the majority of studies assessed fathers’ responses in comparison to mothers or between fathers’ groups, lacking research exploring differences between fathers and non-fathers as controls.

Research on parenting is limited by the complexity and difficulty in its definition, reflected in the variety of methods of assessment of different parental dimensions (e.g., parenting behavior, attitudes, satisfaction, and beliefs, stress; Smith, 2011; Hurley et al., 2014). In our review, measurements ranged from questionnaires or rating scales (e.g., Gettler et al., 2011; Li et al., 2018), to semi-structured interviews (e.g., Feldman et al., 2011; Kim et al., 2015) and father–infant interaction paradigms (i.e., home naturalistic observations or structured observations while undergoing a task), with different composites (e.g., Gordon et al., 2010a; Feldman et al., 2012; Weisman et al., 2013a). Consequently, the difference in operationalization of parenting and quality of parental care may contribute to the observed discrepancies in results.

In addition, another factor that may impact the quality of the available findings across the neurobiological correlates is the consideration of confounding variables. While the majority of the reviewed studies included covariates in their models that have been shown to impact results (e.g., age, diurnal variability of collection of hormone levels) or examined potential correlations with those prior to data analysis, future research should consider other covariables that importantly impact parenting, such as the level of father’s investment in child’s care (individually and inter-correlation of maternal and paternal investment), as well as measures of relationship functioning and satisfaction. Indeed, both relationship effort and parental effort are thought to influence male parental care (Anderson et al., 1999; Hofferth and Anderson, 2003), with parental investment being differently determined by individual and shared couple factors, such as parental wellbeing, marital relationship, and social support (Parke, 2002; Cox et al., 2004; Gameiro et al., 2011). For example, a study by Saxbe et al. (2017) found that hormonal changes (decline in T during pregnancy) and degree of synchrony with mothers (correlations with mothers’ T) predicted father’s paternal investment.

During the postpartum period, endocrine regulation arising from parent–infant interactions may influence complex structural and functional changes in the brain, mediating caregiving responses. Differences in fathers’ hormonal levels during the postpartum period were found to be associated with neural activation to infant stimuli in several brain regions (Atzil et al., 2012; Kuo et al., 2012; Abraham et al., 2014). Overall, the studies reviewed suggest that the experience of fatherhood and exposure to infant cues are associated with neuroplasticity and significant changes in fathers’ hormones and neural responses related to reward, attachment, and emotion processing. Parent–infant interactions may be an important contributor to the neuroendocrine changes, which in turn influence parenting behaviors (Rajhans et al., 2019). However, the current research cannot ascertain the direction of causality present: if changes in neurobiological components lead to differences in parenting repertoires or if the latter contributes to changes in hormonal and brain circuits. The findings highlight the need for more longitudinal experimental research evaluating the association between hormones, brain structure and function, and paternal behaviors.

Although not in the scope of our review (for a review see Rajhans et al., 2019), evidence indicates that mothers and fathers’ experiences are somewhat comparable, with both parents displaying brain activations in parenting-related networks when viewing their own infant and increases in OT (Atzil et al., 2012). However, important differences in parenting behaviors due to neuroendocrine responses to own infant stimuli have been observed (Rajhans et al., 2019). For example, gray matter decreases reported seem unique to fathers (Kim et al., 2014). Overall, the literature suggests that mothers’ changes may be more related to affectionate and warm maternal behavior, while fathers underlie stimulatory and exploratory play paternal behavior (Rajhans et al., 2019). Rajhans et al. (2019) importantly highlight that the type and amount of contact between parent and infant during the early postpartum period may also critically influence parental sex differences, alongside gender-specific biology. This is in accordance with findings observed in endocrine and functional connectivity domains.

Our review provides a broad overview of published primary studies related to neurobiological correlates of fatherhood, updating and extending existing reviews (e.g., Swain, 2011; Feldman, 2015; Swain and Ho, 2017). In our integrative and comprehensive review, we intended to additionally organize a systematic framework for future directions (for a detailed framework for future research in the field, see Table 2). Despite the rigorous methodology, this study is not without limitations. Due to our research question, our inclusion criteria were strict, and we only described changes reported in the defined postpartum window (e.g., studies that did not report results specifically and independently for the 0–12 months postpartum period were not considered). Thus, even though we attempted to have a search strategy as comprehensive as possible, we understand that by skipping the prepartum period we might not have identified all relevant studies in the field. Evidence for changes in fathers during pregnancy has been emerging (e.g., Bakermans-Kranenburg et al., 2019; Diaz-Rojas et al., 2021) and should be considered in future reviews. Also, we did not report results concerning homosexual primary caregiving fathers, due to the possible underlying differences in the organization of care behaviors. We recognize the existence of different family organizations (e.g., adoptive parents), and this study is not representative of this diversity. It is also important to highlight that it is increasingly necessary to actively implement efforts to make research more inclusive in this regard and so we added this vector of interest to Table 2. In addition, for practical reasons, the search was limited to English language. As it is not required for scoping reviews, we did not perform a quality assessment of the included studies. We did not include grey literature as well, as our main aim was focused on published reports. Finally, as we did not perform an updated second search, our results are only up to date as of December 2020.

**Table 2:** Systematic framework for future directions.

Identified gaps in research
Influence of number of children in father’s involvement in childcare and its association with T levels
Direction of the association between PRL levels and the experience of fatherhood
Associations between CORT levels and each dimension of interest (measures of caregiving vs. measures of father–infant interaction) across time
Neurogenetic studies in addition to peripheral measures in OT assessments
Studies evaluating evidence for molecular mechanisms (e.g., epigenetic modifications) related to fatherhood
Cross-sectional and longitudinal studies that investigate the association between neurostructural changes and fatherhood
Association between structural and functional brain changes and its effect in parenthood in fathers
Studies exploring functional brain activity differences between fathers and non-fathers as controls
Longitudinal experimental research evaluating the association between hormones, brain structure and function and paternal behaviors
Further inclusion of different family organizations in research
Inclusion of measures of father’s investment in child’s care (individually and inter-correlation of maternal and paternal investment) and measures of relationship functioning and satisfaction

T, Testosterone; PRL, Prolactin; CORT, Cortisol; and OT, Oxytocin.

Fathers’ parental brain is an emerging area of research. The postpartum period is a critical stage for the development of father–infant interactions and attachment. Across studies, findings have provided strong evidence for neuroplasticity changes in fathers during the postpartum period. The endocrine, structural and functional changes that occur during this period critically contribute to fathers’ caregiving repertoires and the quality of paternal care. The presence of plasticity in the postpartum period indicates that fathers’ brains may be changed by their parenting experiences. Despite the challenges in studying the neurobiological mechanism of paternal behavior due to tissue changes specificities, the studies present in this review highlight the consistency of OT among biological tissues, namely saliva and plasma levels (Feldman et al., 2010a,b, 2011). This finding is of special relevance in the field of attachment neurobiology studies, allowing for non-invasive (i.e., saliva samples) and reliable methodologies in assessing this biological marker in the context of paternal behavior. Future research should continue to longitudinally explore the normative changes associated with the experience of fatherhood across the peripartum period.

In conclusion, the current review highlights the multi-dimensional interplay accounting for parenting behavior, shedding light on the neuroendocrine, molecular, and brain mechanisms with relevance for the understanding of an evolutionary human social behavior.

## Author Contributions

MS, FP, JA, BP, SM, RG, IS, AS, AM and AG-Á: conceptualization. MS, AG-Á, and AM: methodology. AG-Á, RG, and AM: validation. MS, FP, JA, BP, and SM: formal analysis, investigation, and writing-original draft preparation. AG-Á, RG, AM, IS, and AS: writing-review and editing. MS and FP: visualization. AG-Á and AM: supervision. All authors have read and agreed to the published version of the manuscript.

## Funding

AM was supported by the Portuguese Foundation for Science and Technology (FCT) and EU through the European Social Fund and the Human Potential Operational Program – IF/00750/2015. JA was supported by the Psychology Research Centre (UI1662), University of Minho, through an individual Research Fellowship (UMINHO/BIM-CNCG/2021/28), framed by the Multiannual Funding of R&D Units (UIDB/01662/2020), and supported by the Portuguese Foundation for Science and Technology (FCT)/Ministério da Ciência, Tecnologia e Ensino Superior (MCTES) through national funds (PIDDAC). RG, BP, and MS are supported by a Ph.D. Grant (SFRH/BD/5099/2020; 2020.10167.BD; 2021.07006.BD, respectively) and sponsored by the Portuguese Foundation for Science and Technology. AG-Á is supported by the Portuguese Foundation for Science and Technology [Individual Call to Scientific Employment Stimulus – 3rd Edition 2019 – 2020.02059.CEECIND]. AM, AS, and IS are also supported by the Psychology Research Centre (PSI/01662), School of Psychology, University of Minho, through the Portuguese Foundation for Science and Technology by the Portuguese State Budget (Ref.: UIDB/PSI/01662/2020). The Center for Research in Neuropsychology and Cognitive and Behavioral Intervention (CINEICC) of the Faculty of Psychology and Educational Sciences of the University of Coimbra is supported by the Portuguese Foundation for Science and Technology and the Portuguese Ministry of Education and Science through national funds and co-financed by FEDER through COMPETE2020 under the PT2020 Partnership Agreement [UID/PSI/01662/2013].

## Conflict of Interest

The authors declare that the research was conducted in the absence of any commercial or financial relationships that could be construed as a potential conflict of interest.

## Publisher’s Note

All claims expressed in this article are solely those of the authors and do not necessarily represent those of their affiliated organizations, or those of the publisher, the editors and the reviewers. Any product that may be evaluated in this article, or claim that may be made by its manufacturer, is not guaranteed or endorsed by the publisher.

## Footnotes

^1^ https://osf.io/j2x9u
