# Supplementary material for: Neurobiological Correlates of Fatherhood During the Postpartum Period: A Scoping Review
Source: Front Psychol. 2022 Feb 3;13:745767. doi: 10.3389/fpsyg.2022.745767 (PMC8850250; doi:10.3389/fpsyg.2022.745767)
Supplement: Supplementary file 1 [file Table_1.DOCX]

Supplementary Material

# Search Strategy for the Included Databases

| Database | Search Strategy | Results |
| --- | --- | --- |
| PubMed | (((((((((((((((((((father OR fatherhood OR parent OR dad) AND (postpartum OR postnatal OR perinatal)) AND ("human"[All Fields])) AND ((Neuronal plasticity OR anatomical OR structural OR gray matter volume OR cortical thickness OR functional OR connectivity OR activation OR resting state OR testosterone OR oxytocin OR genetic OR genes OR epigenetic OR molecular OR molecular mechanisms OR neuroimaging))) AND (English[Language]))) AND (("2005"[Date - Publication] : "2020"[Date - Publication])) AND (2005:2020[pdat])) NOT ("depression"[Title/Abstract])) NOT ("intervention"[Title/Abstract])) ) NOT ("disorder"[Title/Abstract])) NOT ("schizophrenia"[Title/Abstract])) NOT ("alcohol"[Title/Abstract])) NOT ("monkey"[Title/Abstract])) NOT ("mice"[Title/Abstract])) NOT ("milk"[Title/Abstract])) NOT ("disease"[Title/Abstract])) NOT ("monkeys"[Title/Abstract])) NOT ("smoking"[Title/Abstract])) NOT ("breastfeeding"[Title/Abstract]) | 545 |
| Scopus | ALL ( ( father OR fatherhood OR parent OR dad ) AND ( cerebral AND cortex OR neuronal AND plasticity OR brain OR anatomical OR structural OR gray AND matter AND volume OR cortical AND thickness OR functional OR connectivity OR activation OR resting AND state OR hormonal OR hormones OR neuroendocrine OR testosterone OR oxytocin OR genetic OR genes OR epigenetic OR molecular OR molecular AND mechanisms OR neuroimaging OR mri OR fmri OR functional AND neuroimaging ) AND ( postpartum OR postnatal OR perinatal ) AND ( human ) ) AND PUBYEAR > 2004 AND LANGUAGE ( english ) AND ( LIMIT-TO ( SRCTYPE , "j" ) ) AND ( LIMIT-TO ( DOCTYPE , "ar" ) OR LIMIT-TO ( DOCTYPE , "re" ) ) AND ( LIMIT-TO ( LANGUAGE , "English" ) ) | 886 |
| PsycINFO | 1 ((Father or fatherhood or parent or dad) and (cerebral cortex or Neuronal plasticity or Brain or anatomical or structural or gray matter volume or cortical thickness or functional or connectivity or activation or resting state or hormonal or hormones or neuroendocrine or testosterone or oxytocin or genetic or genes or epigenetic or molecular or molecular mechanisms or neuroimaging or MRI or FMRI or functional neuroimaging) and (postpartum or postnatal or perinatal) and human).mp. [mp=title, abstract, heading word, table of contents, key concepts, original title, tests & measures, mesh]  2 limit 1 to (human and yr="2005 -Current") | 42 |
